# Inhibitory effect and mechanism of action of juniper essential oil on gray mold in cherry tomatoes

**DOI:** 10.3389/fmicb.2022.1000526

**Published:** 2022-09-23

**Authors:** Yu-Xuan Wu, Yun-Di Zhang, Na Li, De-Dong Wu, Qi-Meng Li, Yun-Ze Chen, Guo-Cai Zhang, Jing Yang

**Affiliations:** ^1^Heilongjiang Province Key Laboratory of Forest Protection, School of Forest, Northeast Forestry University, Harbin, China; ^2^School of Biological Sciences, Guizhou Education University, Guiyang, China; ^3^College of Forestry, Guizhou University, Guiyang, China

**Keywords:** juniper essential oil, antifungal activity, *Botrytis cinerea*, cherry tomato, gray mold, enzyme activity, GC-MS

## Abstract

Juniper essential oil (JEO), which is mostly known as an immune system booster and effective detoxifier, has substantial antimicrobial activity. A comparison of the inhibitory effects of three plant essential oils from juniper (*Juniperus rigida*), cedarwood (*Juniperus virginiana*), and cypress (*Crupressus sempervirens*) on four plant pathogenic fungi indicated that JEO was the most effective at inhibiting the growth of gray mold (*Botrytis cinerea*). Additional studies were subsequently conducted to explore the *in vivo* and *in vitro* antifungal activity and possible mechanism of JEO against *B. cinerea*. The results show that JEO inhibited the germination of spores and mycelial growth of *B. cinerea* in a concentration-dependent manner and exhibited strong inhibition when its concentration exceeded 10 μL/mL. JEO also significantly inhibited the incidence of disease and diameters of gray mold lesions on cherry tomato fruit (*Solanum lycopersicum*). After 12 h of treatment with JEO, the extracellular conductivity, and the contents of soluble protein, malondialdehyde, and hydrogen peroxide were 3.1, 1.2, 7.2, and 4.7 folds higher than those of the control group, respectively (*P* < 0.05), which indicated that JEO can damage membranes. Scanning electron microscopy observations revealed that JEO affected the morphology of mycelia, causing them to shrivel, twist and distort. Furthermore, JEO significantly improved the activities of the antioxidant-related enzymes superoxide dismutase and catalase but reduced the pathogenicity-related enzymes polygalacturonase (PG), pectin lyase and endoglucanase of *B. cinerea* (*P* < 0.05). In particular, PG was reduced by 93% after treatment with JEO for 12 h. Moreover, the 18 constituents of JEO were identified by gas chromatography/mass spectrometry (GC-MS) analysis, mainly limonene (15.17%), γ-terpinene (8.3%), β-myrcene (4.56%), terpinen-4-ol (24.26%), linalool (8.73%), α-terpineol (1.03%), o-cymene (8.35%) and other substances with antimicrobial activity. Therefore, JEO can be an effective alternative to prevent and control gray mold on cherry tomato fruit.

## Introduction

Cherry tomato fruit (*Solanum lycopersicum*) are popular worldwide owing to their color, taste, and high levels of nutrients (Guo et al., 2021). However, as a climacteric fruit, they have a short postharvest life, and they are also vulnerable to environmental factors and phytopathogenic fungi that can cause the fruit to decay and deteriorate (Raynaldo et al., 2021). *B. cinerea* (gray mold) can infect all the organs of tomato except the roots, causing lesions in cherry tomatoes before and after picking, thus, resulting in reduced tomato yields (Guerra et al., 2015; Álvarez et al., 2021). In addition, *B. cinerea* can overwinter in the soil as spores or hyphae and reinfect the following year (Rguez et al., 2020). Gray mold frequently occurs in tomatoes during postharvest transport and storage, affecting the postharvest and shelf life of the fruit (Wang et al., 2010). Although synthetic fungicides do not deleteriously affect the appearance of fruit and can effectively control the occurrence of cherry tomato diseases, the excessive use of synthetic fungicides can lead to pesticide residues in the fruit and increased resistance to pathogenic fungi, endangering human health and environmental safety (Romanazzi et al., 2016). Therefore, it is necessary to find an environmentally friendly fungicide that can be used to sustainably control the occurrence of gray mold on cherry tomatoes (Touba et al., 2012; Ji et al., 2018).

Plant essential oils (PEOs) are volatile compounds that are extracted from various organs of plants through distillation and extraction. Many studies have shown that they can have a wide range of insecticidal, antibacterial, antifungal, antioxidant (Raut and Karuppayil, 2014), and anticancer properties (Hong et al., 2022). For example, the essential oil from *Pulicaria crispa* has an antibacterial effect on Gram-positive bacteria (AlMotwaa and Al-Otaibi, 2022). *Ziziphora clinopodioides* essential oil (ZCEO) inhibits mycelial growth and spore germination of *Sclerotinia sclerotiorum* (Ma et al., 2016). The essential oil from *Origanum vulgare* has an inhibitory effect on *B. cinerea* (Zhao et al., 2021). In addition, cinnamon oil, origanum oil, fennel oil and thyme oil have been found to inhibit *Fusarium oxysporum* occurred in strawberries (Park et al., 2017). The US Food and Drug Administration (FDA) has classified some PEOs as safe for human consumption because they cause less harm to the environment and human health (Hu et al., 2019; Bajac et al., 2022). Morales-Rabanales et al. (2022) found that a complex membrane of *Schinus mole* essential oil and chitosan inhibited the infestation of tomato fruit by *Fusarium oxysporum* f. sp. *lycopersici*.

*Juniperus rigida* Sieb. et Zucc., the temple juniper, is widely distributed in temperate regions of the northern hemisphere (Ghorbanzadeh et al., 2021). It is native to northern China, Korea and Japan, and is used in traditional Tibetan and Mongolian medicine to treat rheumatoid arthritis, edema, coughs, and skin diseases (Kim et al., 2010). Juniper extracts are known to contain phenolic substances, including flavonoids, chlorogenic acid, and catechin, terpenoids, podophyllotoxin, and other active substances that possess antibacterial, anticancer, and antioxidant activities (Gordien et al., 2009; Feng et al., 2020). Juniper essential oil (JEO) also contains a large number of terpenes, including α-pinene, limonene, β-pinene, and β-laurene (Zheljazkov et al., 2017). Although there is slight variation in the composition of the essential oil extracted from juniper in different regions, it is highly effective at inhibiting the human bacterial pathogens *Clostridium perfringens* and *Staphylococcus aureus* and the pathogenic yeast *Candida clabrata* (Zheljazkov et al., 2018).

Recently, there has been a substantial amount of study on the methods of extraction, determination of the chemical components and antioxidant effects of JEO, but there are few studies on whether JEO inhibits the growth of phytopathogenic fungi. In this study, the inhibitory effects of three plant essential oils, including juniper (*Juniperus rigida*) essential oil (JEO), cedarwood (*Juniperus virginiana*) essential oil (CWEO), and cypress (*Cupressus sempervirens*) essential oil (CPEO), on *B. cinerea*, *F. oxysporum*, *Pestalotiopsis neglecta*, and *Alternaria alternata* were compared. JEO was the most effective at inhibiting gray mold *in vitro*. Thus, the ability of JEO to control gray mold in cherry tomato fruit *in vivo* was explored. Moreover, the possible mechanism of action involved was investigated. This study provides a theoretical basis for the wider application of JEO to prevent and control gray mold in cherry tomato and possibly even other plant diseases.

## Materials and methods

### Materials

All the unilateral essential oils, including JEO, CWEO, and CPEO, were purchased from Jiangxi Yisenyuan Plant Fragrance Co., Ltd. (Nanchang, China). Test kits of malondialdehyde (MDA) content, hydrogen peroxide (H_2_O_2_) content, superoxide dismutase (SOD), catalase (CAT), polygalacturonase (PG), pectin lyase (PL), and endo-β-1-4 glucanase (EG) enzyme activity were obtained from Suzhou Grace Biotechnology Co., Ltd., (Suzhou, China). Other chemicals were analytical grade or better, purchased from Fuyu Fine Chemical Co., Ltd. (Tianjin, China).

Healthy and mature fresh cherry tomatoes were picked at the Kaiyang Lake Picking Orchard in Harbin, Heilongjiang Province, China.

### Pathogen and cultures

The phytopathogenic fungi *B. cinerea, F. oxysporum*, *P. neglecta*, and *A. alternata* were provided by the Heilongjiang Province Key Laboratory of Forest Protection (Northeast Forestry University, Harbin, China) and cultured on potato dextrose agar (PDA) plates at 25°C.

The spore suspensions of *B. cinerea* were harvested and filtered through sterilized cotton as described by Ji et al. (2018). The spore concentrations were adjusted to 1 × 10^6^ spores/mL using a hemocytometer under 400 × magnification *via* inversed optical microscopy.

### Mycelial growth inhibition

The inhibitory effects of the three essential oils against the mycelial growth of *B. cinerea*, *F. oxysporum*, *P. neglecta*, and *A. alternata* were determined as described by Xu et al. (2021) with slight modifications. Ten percent Tween 80 (v/v) was used as a cosolvent to add JEO, CWEO and CPEO to the PDA media at final concentrations of 0, 2.5, 5, 10, 20, and 40 μL/mL for each treatment. Mycelial plugs (5 mm in diameter) taken from 7-day-old fungal cultures were placed at the center of 60 mm Petri dishes, and the plates were then sealed and placed in a 25°C incubator in the dark. The percentage of mycelial growth was calculated after 5 d by measuring the radial growth diameter. Each treatment was performed in triplicate.

### Effect of juniper essential oil on the spore germination of *Botrytis cinerea*

The hanging drop method (Qiu et al., 2014) was used to assess the effect of JEO on the germination of *B. cinerea* spores. A suspension of 20 μL of spores was mixed with 20 μL liquid medium potato dextrose broth (PDB) that contained JEO to final concentrations of 0, 1.25, 2.5, 5, 10, 20, and 40 μL/mL. A volume of 40 μL was added to the hemocytometer. The percentage of spore germination was recorded after incubation at 25°C for 12 h. Each treatment was conducted in triplicate, and five fields of view were randomly selected for microscopic examination in each replicate.

### Effect of juniper essential oil at controlling gray mold in cherry tomato fruit

Fresh and healthy cherry tomato fruit were selected. Their surfaces were disinfected with 0.05% sodium hypochlorite for 2 min, and then rinsed with sterile distilled water 3 times for 30 s each time. The fruit were punctured in the middle (2 mm in diameter and 3 mm in depth) with a sterile stainless steel needle. A volume of 10 μL of JEO at concentrations of 40, 80, and 160 μL/mL were added to the wounds, while the control group was treated with Tween 80. Each concentration was considered as a different treatment, contained 10 tomatoes, and was repeated three times. The treated fruits were air-dried for 4 h, and 10 μL of spore suspension was added to the wound. They were then placed in 160 mm Petri dishes covered with plastic wrap and incubated at 25°C. After 4 d, the rate of incidence rate of the infection of cherry tomatoes was recorded, and the diameter of the lesions was determined using a Vernier caliper (Jiao et al., 2020).

### Effects of juniper essential oil on extracellular conductivity and soluble protein leakage

Five mycelial plugs of *B. cinerea* (diameter 5 mm) were placed in 180 mL of PDB media and cultured shaking at 150 rpm at 25°C for 48 h. Add 20 mL of a mixture of JEO and Tween 80, and the cultivation was continued. The JEO content was 1.1216 and 0.865 mL respectively, and the final medication-containing medium concentrations were 5.608 μL/mL (EC_50_) and 4.325 μL/mL (EC_30_). The control group contained an equal amount of Tween 80. Mycelial samples and supernatant were collected 3, 6, 9, and 12 h after dosing. The mycelia were filtered through a sterile silk cloth, rinsed three times with 0.9% NaCl, and dried with sterile filter paper for later use. A DDS-11 digital conductivity meter (Shanghai Precision Scientific Instrument Co., Ltd., Shanghai, China) was used to measure the extracellular conductivity (μS/cm). Soluble protein leakage was measured as described by Bradford (1976). Each treatment was conducted in triplicate.

### Effects of juniper essential oil on the contents of malondialdehyde and hydrogen peroxide

The membrane damage of *B. cinerea* owing to exposure to JEO was assessed in more detail by measuring the contents of MDA and hydrogen peroxide (H_2_O_2_). The mycelia obtained were processed following the kit instructions and measured using an N600 double-beam UV/Vis spectrophotometer (Shanghai Ruckus Instrument Co., Ltd., Shanghai, China). Each treatment was conducted in triplicate.

### Effect of juniper essential oil on enzyme activities

The mycelia and supernatant were collected as described above and processed following the kit instructions. The enzyme activities including superoxide dismutase (SOD), catalase (CAT), polygalacturonase (PG), pectin lyase (PL), and endo-β-1-4-glucanase (EG) were determined according to the manufacturer’s instructions, using an N600 dual-beam UV/Vis spectrophotometer.

### Mycelial morphology observation

A scanning electron microscope (SEM) was used to study the effect of JEO on mycelial morphology during the growth of *B. cinerea*. One disc (7.5 mm in diameter) of the mycelial plug was inoculated into PDA media that contained 0 and 5.608 μL/mL JEO. A sterilized coverslip was then inserted at a 45° angle at 2–3 cm from the mycelial plug and cultured upside down for 5 d. After the mycelia grew on the coverslip, the samples were processed as described by Li et al. (2017b) with slight modifications and observed at 10,000 × magnification under an SEM.

### Gas chromatography/mass spectrometry analysis

The composition of JEO was analyzed by GC-MS, JEO diluted 1:100 with hexane, Model 7890A–7000B (Agilent Technologies Inc., USA). Helium was used as the carrier gas at a flow rate of 1 mL/min, with a split ratio of 1:40 using an HP-5MS (30 × 0.25 mm id, 0.25μm film thickness) capillary column, the temperature at the inlet was 250°C. The GC column temperature was programmed as follows: Initial temperature 60°C, hold for 1 min, increase to 250°C at 3°C/min for 5 min. Ionization voltage: 70 eV, full scan.

### Statistical analysis

All the experiments were performed in triplicate. Microsoft Excel 2019 (Redmond, WA, USA) was used to calculate the mean values and standard deviations (*n* = 3). The effective concentration that inhibited 50% of the mycelial growth (EC_50_) and its corresponding 95% confidence limits were estimated by Probit analysis using SPSS 26.0 (IBM, Inc., Armonk, NY, USA). In addition, a one-way analysis of variance (ANOVA) was performed, and statistical significance was set as *P* < 0.05. Origin 9.8 (OriginLab, Inc., Northampton, MA, USA) and Microsoft Publisher 2010 (Redmond, USA) were used to prepare the graphics.

## Results

### Juniper essential oil, cedarwood essential oil and cypress essential oil inhibit the mycelial growth of phytopathogenic fungi

**FIGURE 1:**
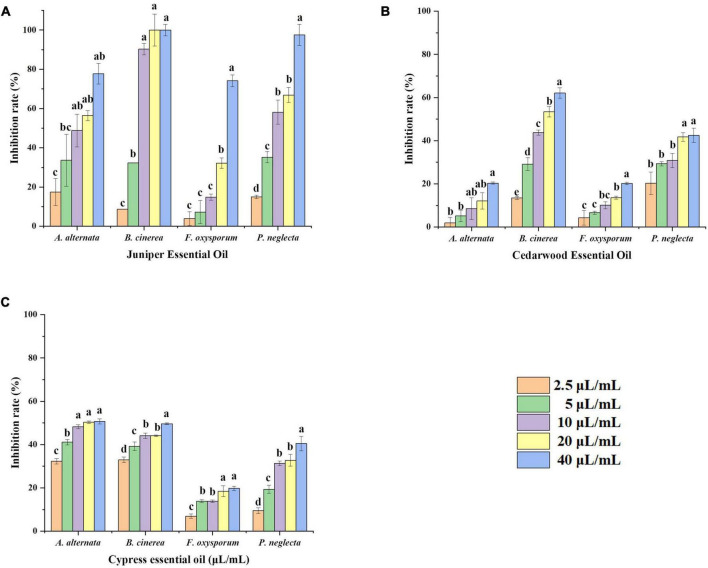
Effect of different concentrations of plant essential oils on the rate of inhibition of mycelial growth of four pathogenic fungi. Mycelial growth inhibition rates were calculated for JEO **(A)** CWEO **(B)**, and CPEO **(C)** after treatment with different concentrations (0, 2.5, 5, 10, 20, and 40 μL/mL) of plant essential oils and incubated for 5 d at 25°C. Bars represent the standard error of the mean (*n* = 3). Letters (a, b, c, d) indicate statistically significant differences between concentrations according to Duncan’s multiple range test (*P* < 0.05). CPEO, cypress essential oil; CWEO, cedar wood essential oil; JEO, juniper essential oil.

Compared with the control groups, the growth of fungi was inhibited in the three essential oils. The inhibitory effects of JEO, CWEO, and CPEO on the mycelial growth of phytopathogenic fungi are shown in Figure 1. The three essential oils inhibited the mycelial growth in a concentration-dependent manner, and there was a significant difference between the effects of essential oils at high concentrations (40 μL/mL) and low concentrations (2.5 μL/mL) (*P* < 0.05). In addition, different agents at the same concentration have varying inhibitory effects on pathogens. For example, 40 μL/mL of the three essential oils inhibited *F. oxysporum* by 74.2, 20.2, and 19.8%, respectively. The EC_50_ values and the virulence regression equation of the three essential oils against the four phytopathogenic fungi are shown in Table 1.

**TABLE 1:** Toxicity equations for juniper essential oil, cedarwood essential oil, and cypress essential oil against *Alternaria alternata*, *Botrytis cinerea*, *Fusarium oxysporum*, and *Pestalotiopsis neglecta.*

Essential oil	Phytopathogenic fungi	Toxicity equation	χ^2^	Correlation coefficient	EC_50_ (mg/mL)	95% confidence interval (mg/mL)
Juniper Essential Oil	*A. alternata*	Y = 1.307X-1.4	2.098	0.979	11.784	9.597–14.658
	*B. cinerea*	Y = 4.647X-3.68	6.625	0.967	5.608	4.481–7.040
	*F. oxysporum*	Y = 2.130X-3.006	11.044	0.932	25.753	16.227–65.261
	*P. neglecta*	Y = 2.016X-1.853	12.108	0.924	8.297	4.602–13.920
Cedarwood Essential Oil	*A. alternata*	Y = 0.941X-2.346	0.307	0.984	310.514	117.986–3223.594
	*B. cinerea*	Y = 1.118X-1.391	2.554	0.961	17.527	13.725–23.836
	*F. oxysporum*	Y = 0.722X-2.008	0.057	0.997	601.887	157.410–32635.050
	*P. neglecta*	Y = 0.532X-0.985	1.219	0.927	71.25	33.22–590.632
Cypress Essential Oil	*A. alternata*	Y = 0.391X-0.53	1.529	0.851	22.715	11.741–161.471
	*B. cinerea*	Y = 0.327X-0.530	0.456	0.93	41.956	17.258–16094.669
	*F. oxysporum*	Y = 0.454X-1.528	1.325	0.853	2309.951	237.443–1.919E9
	*P. neglecta*	Y = 0.805X-1.464	3.08	0.914	66.032	38.109–193.932

### Effect of juniper essential oil on the spore germination of *Botrytis cinerea*

**FIGURE 2:**
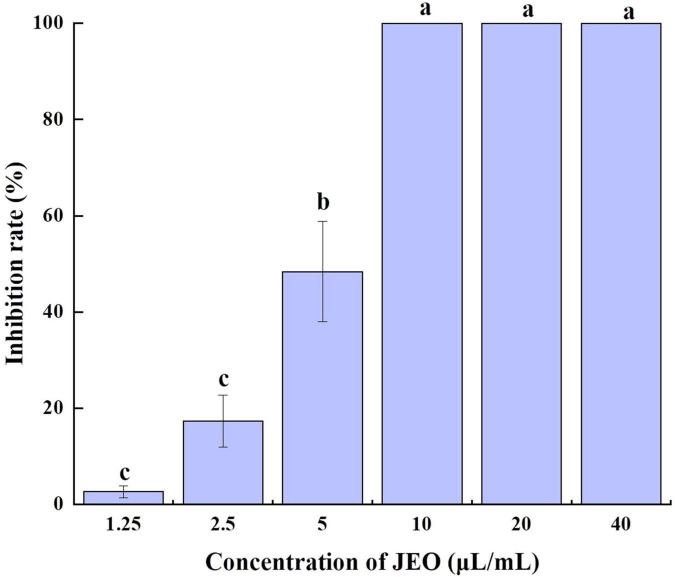
Effects of different concentrations of JEO on the spore germination of *B. cinerea*. The spores were treated with JEO at different concentrations (0, 1.25, 2.5, 5, 10, 20, and 40 μL/mL) and then incubated at 25 °C for 12 h. The bars represent the standard error of the mean (*n* = 3). Letters (a, b, c, d) represent statistically significant differences between different concentrations according to Duncan’s multiple range test (*P* < 0.05). JEO, juniper essential oil.

The germination of *B. cinerea* spores was significantly inhibited by JEO at different concentrations (*P* < 0.05) as shown in Figure 2. After incubation for 12 h, 90% of the spores in the control group had germinated. The germinating spores in the 1.25 μL/mL JEO treatment were inhibited by 2.64%, while the spore germination was completely inhibited at concentrations of 10–40 μL/mL.

### Ability of juniper essential oil to control gray mold in cherry tomato fruit

**FIGURE 3:**
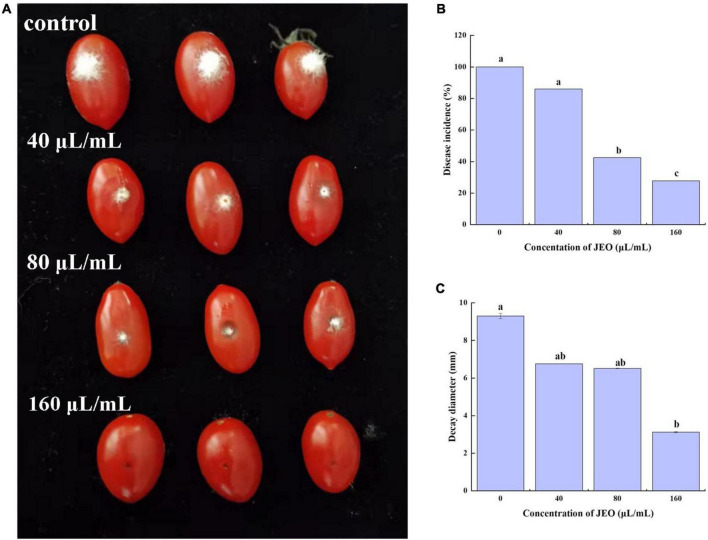
Effect of JEO on infection with *B. cinerea* in cherry tomato fruits after harvest. Each cherry tomato was inoculated with a 10 μL spore suspension of *B. cinerea* at 1 × 10^6^ spores/mL and then treated with JEO at different concentrations (0 as control, 40, 80, and 160 μL/mL) for 4 d at 25°C. The representative photographs **(A)**, disease incidence **(B)** and decay diameter **(C)** of the cherry tomatoes were recorded on day 4 after inoculation. The bars represent the standard error of the mean (*n* = 3). Letters (a, b, c, d) represent statistically significant differences between different concentrations according to Duncan’s multiple range test (*P* < 0.05). JEO, juniper essential oil.

As shown in Figure 3, JEO inhibits the lesion diameters or disease incidence of gray mold caused by *B. cinerea* on cherry tomato fruit. The incidence of gray mold on cherry tomato fruit was significantly reduced (*P* < 0.05) when the amount of JEO was increased from 0 (control) to 160 μL/mL. However, there was no significant difference in lesion diameter after treatment with 40 and 80 μL/mL of JEO, respectively. The disease incidence and lesion diameter were reduced by 72.2 and 66.4% in the 160 μL/mL JEO treatment group, respectively.

### Effects of juniper essential oil on extracellular conductivity and soluble protein leakage

**FIGURE 4:**
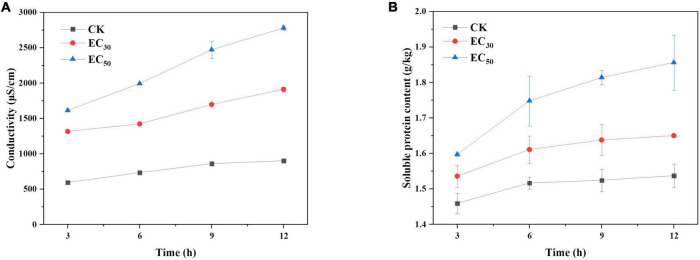
Effect of JEO on extracellular conductivity **(A)** and soluble protein content **(B)** of *B. cinerea* mycelia during 12 h of incubation. *B. cinerea* was treated with different concentrations of JEO (0 as control, EC_30,_ and EC_50_). The bars represent the standard error of the mean (*n* = 3).

As shown in Figure 4A, the value of extracellular conductivity in the control group was maintained at a low level for 12 h despite a slight increase. However, the values for the treatment group with JEO increased significantly with time (*P* < 0.05). The conductivity value of the EC_50_ treatment was 1.45- and 3.1-fold higher than that of the EC_30_ and control group, respectively, after 12 h of incubation. The data indicated that JEO can damage the cell membranes of *B. cinerea.*

The effect of JEO on soluble protein leakage of *B. cinerea* is shown in Figure 4B. The trend was similar to the extracellular conductivity in a time- and concentration-dependent manner. The content of soluble protein was significantly higher than that of the control group after the EC_50_ treatment for 12 h (*P* < 0.05), indicating that a high concentration of JEO aggravated the leakage of soluble proteins of *B. cinerea*.

### Effects of juniper essential oil on the contents of malondialdehyde and hydrogen peroxide

**FIGURE 5:**
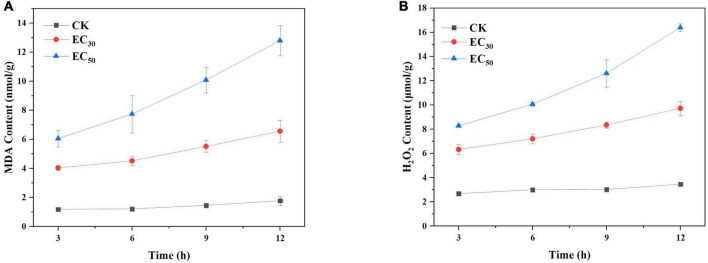
Effects of JEO on MDA content **(A)** and H_2_O_2_ content **(B)** from *B. cinerea* mycelia during 12 h of incubation. The mycelia were treated with different concentrations of JEO (0 as control, EC_30_, and EC_50_). The bars represent the standard error of the mean (*n* = 3). JEO, juniper essential oil; MDA, malondialdehyde.

MDA and H_2_O_2_ are major markers for the peroxidation of membrane lipids. The contents of MDA and H_2_O_2_ in *B. cinerea* treated with JEO are shown in Figure 5. They were relatively low and had changed little in the control group. However, the treatment groups were significantly higher than those of the control group (*P* < 0.05) in a time-dependent manner. The contents of MDA and H_2_O_2_ of *B. cinerea* in the EC_50_ treatment were 7.2- and 4.7-fold higher than those of the control group, respectively, after 12 h incubation.

### Effect of juniper essential oil on the enzyme activity of *Botrytis cinerea*

#### Effects of juniper essential oil on the activity of antioxidative-related enzymes of *Botrytis cinerea*

**FIGURE 6:**
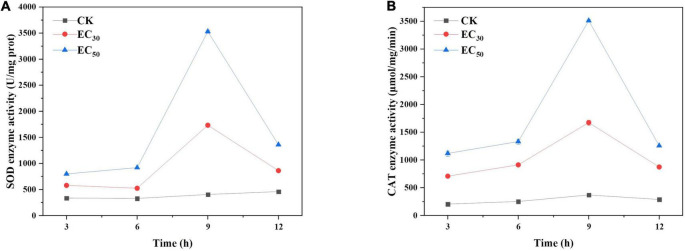
Effects of JEO on the activities of CAT **(A)** and SOD **(B)** secreted from *B. cinerea* mycelia during 12 h of incubation. *B. cinerea* was treated with different concentrations of JEO (0 as control, EC_30_, and EC_50_). The bars represent the standard error of the mean (*n* = 3). CAT, catalase; JEO, juniper essential oil; SOD, superoxide dismutase.

As shown in Figure 6, the SOD and CAT activities in *B. cinerea* treated with JEO were significantly higher than those in the control groups and tended to increase followed by a tendency to decrease within 12 h. The maximum values occurred at 9 h and were significantly higher than those at other times (*P* < 0.05). The SOD and CAT of *B. cinerea* treated with EC_50_ were 9.6- and 9.5-fold higher than those of the control at 9 h, respectively.

#### Effect of juniper essential oil on the pathogenic enzyme activities of *Botrytis cinerea*

**FIGURE 7:**
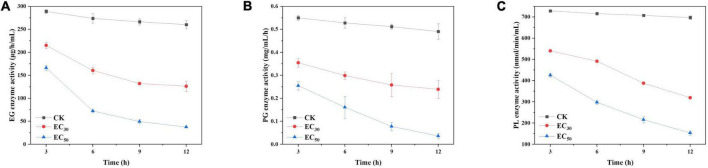
Effects of JEO on the enzyme activities of EG **(A)**, PG **(B)**, and PL **(C)** secreted from *B. cinerea* following treatment with different concentrations of JEO (0 as control, EC_30,_ and EC_50_). The bars represent the standard error of the mean (*n* = 3). EG, endoglucanase; JEO, juniper essential oil; PG, polygalacturonase; PL, pectin lyase.

The activities of enzymes related to pathogenicity (EG, PG, and PL) were significantly lower than those of the control groups following treatment with JEO (*P* < 0.05), respectively (Figure 7). In addition, they decreased gradually as the time of treatment was prolonged. Compared with the control group, the activities of EG, PG and PL decreased by 51.5 and 85.6%; 52.1 and 93%; and 54.2 and 87% following treatment with the EC_30_ and EC_50_ of JEO at 12 h, respectively.

### Effects of juniper essential oil on mycelial morphology

**FIGURE 8:**
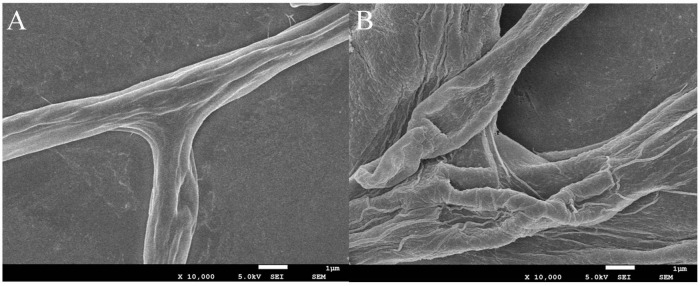
Effect of JEO on the morphology of *B. cinerea*. Mycelia treated with sterilized distilled water as the control **(A)** and EC_50_ of JEO **(B)** were observed by scanning electron microscopy (SEM) at 10,000× magnification. JEO, juniper essential oil.

To explore whether the mycelial morphology of *B. cinerea* was affected by JEO (0 and EC_50_), SEM was utilized to evaluate the morphological alteration. Observation at 10,000 × magnification showed that the untreated hyphae were shaped normally, uniformly thick, smooth on their surface, intact, and undamaged (Figure 8A). However, the mycelia were severely shrunken, distorted, uneven in thickness, and broken after treatment with the EC_50_ of JEO (Figure 8B).

### Gas chromatography/mass spectrometry analysis of the active components in juniper essential oil

The composition of JEO was identified by GC-MS analysis. Eighteen compounds were identified by comparing the mass spectra, relative molecular masses, retention times, and molecular formulae with the NIST library (Table 2). Eighteen compounds accounted for 93.99% of the total content, with terpenes as the main component (51.02%), including limonene (15.71%), 2-carene (10.94%),γ-Terpinene (8.3%), β-Myrcene (4.56%), α-Phellandrene (4.13%), Terpinolene (0.12%), 3-carene (0.07%), α-pinene (0.06%), Humulene (0.06%), Longifolene (0.13%), Camphene (3.89%) and (+)-2-Bornanone (3.05%). This was followed by higher levels of alcohols (34.62%) and alkanes (8.35%) including terpinen-4-ol (24.26%), linalool (8.73%), α-terpineol (1.03%), dl-menthol (0.49%), isoborneo (0.11%), o-cymene (8.35%).

**TABLE 2:** GC-MS analysis of JEO.

No	RT (min)	Compound	Molecular formula	Molecular weight	Peak
					area%
1	5.462	α-Pinene	C_10_H_16_	136.23	0.06
2	6.051	Camphene	C_10_H_16_	204.35	3.89
3	7.271	β-Myrcene	C_10_H_16_	136.23	4.56
4	7.727	α-Phellandrene	C_10_H_16_	136.23	4.13
5	8.176	Terpinen-4-ol	C_10_H_18_O	154.25	24.26
6	8.579	O-Cymene	C_10_H_14_	204.35	8.35
7	8.746	Limonene	C_10_H_16_	136.23	15.71
8	9.259	3-Carene	C_10_H_16_	136.23	0.07
9	9.662	γ-Terpinene	C_10_H_16_	136.23	8.3
10	10.608	Terpinolene	C_10_H_16_	136.23	0.12
11	10.791	2-Carene	C_10_H_16_	136.23	10.94
12	11.274	Linalool	C_10_H_18_O	154.25	8.73
13	12.912	(+)-2-Bornanone	C_10_H_16_O	152.23	3.05
14	13.836	Isoborneol	C_10_H_18_O	154.25	0.11
15	14.113	dl-Menthol	C_10_H_20_O	156.27	0.49
16	14.862	α-Terpineol	C_10_H_18_O	154.25	1.03
17	23.726	Longifolene	C_15_H_24_	204.35	0.13
18	25.714	Humulene	C_15_H_24_	204.35	0.06

## Discussion

Juniper has a wide range of medicinal and commercial values as a renewable resource (Kuang et al., 2019). JEO is a collection of natural compounds extracted from the needles of juniper and is primarily composed of terpenes, similar in chemical composition to juniper berry essential oil (JEBO), which has antibacterial and antioxidant properties (Pandey et al., 2018; Bajac et al., 2022). JEO can inhibit both Gram-positive (*Bacillus cereus* and *B. subtilis*) and Gram-negative bacteria (*Pseudomonas aeruginosa* and *Salmonella typhimurium*) (Falcão et al., 2018). In addition, studies have found that JEO can inhibit *Klebsiella pneumoniae*, leading to protein leakage and cellular deformation (Meng et al., 2016). JEO is widely available, more accessible and has a greater value for exploitation. Therefore, investigating the inhibitory effect of JEO on pathogenic fungi and its possible mechanism provides theoretical support for the additional development of JEO as an environmentally friendly antifungal agent.

In this study, all three PEOs showed different degrees of fungal inhibitory activity, but JEO was the most effective at inhibiting *B. cinerea*. JEO effectively inhibited mycelial growth, spore germination, and the incidence of the gray mold of cherry tomatoes in a concentration-dependent manner. These results are similar to the *in vitro* and *in vivo* inhibition of *B. cinerea* caused by sodium pheophorbide a (SPA) and tea tree oil (TTO) (Li et al., 2017a; Ji et al., 2020).

The cell membrane acts as a barrier to protect the cell by regulating the exchange of compounds inside and outside of the cell and maintaining the stability of the intracellular environment (Tao et al., 2014b). It is the main target for the action of PEOs. For example, the essential oil of mandarin orange (*Citrus reticulata*) disrupted the permeability of the cell membranes of *Penicillium italicum* and *P. digitatum*, leading to the leakage of cell contents and an increase in extracellular conductivity and total lipid content (Tao et al., 2014a). Mint and thyme essential oils reduce the ability of *Rhizopus stolonifer* to infect strawberries by disrupting cell membrane integrity and causing membrane lipid peroxidation (Yan et al., 2021). JEO treatment of *Klebsiella pneumoniae* causes the cells to implode and leak proteins and nucleic acids (Meng et al., 2016). Consistent with the results of this study, JEO similarly increased the extracellular conductivity and soluble protein content of *B. cinerea*, indicating that JEO disrupts the cell membrane integrity of *B. cinerea*.

MDA and H_2_O_2_ are key regulators of the oxidative stress response, and their levels are important parameters that reflect the potential antioxidant capacity of an organism, as well as the degree of cell membrane damage. The terpenoids cembratrien-diols and hinokitiol have been shown to cause an increase in MDA content and induce membrane lipid peroxidation in *B. cinerea* (Wang et al., 2020; Yang et al., 2020). SEM observations revealed that JEO caused alterations in the ultrastructure of the mycelia of *B. cinerea*, with crumpled and broken mycelial surfaces. Relevant studies suggest that JEO may inhibit *B. cinerea* by disrupting its cell membranes (Xu et al., 2021).

In addition, the activities of antioxidant-related enzymes SOD and CAT of *B. cinerea* increased significantly after 9 h of treatment with JEO but decreased at 12 h. This indicates that JEO causes some degree of oxidative stress in *B. cinerea* and has antioxidant capacity. This corresponds to the findings of Zheljazkov et al. (2017). However, the cell wall degrading enzymes (PL, PG, and EG) of *B. cinerea* gradually decreased with increasing time after treatment with JEO. The cell wall degrading enzymes (CWDEs)including cellulase (CL), PG, PL help the pathogenic fungus invade their host by degrading pectin and cellulose, which causes the plant cell walls to rupture (Chinisaz et al., 2014; Zhang et al., 2021). A decrease in the enzyme activities of CWDEs indicates a reduction in the ability of pathogenic fungi to infect their hosts. Yang et al. (2021) found that the pathogenicity-related enzyme activity of both *B. cinerea* and *A. alternata* decreased after treatment with vanillin. Similarly, PG activity was lower in *P. neglecta* treated with SPA than that in the control (Yang et al., 2019), which was consistent with the results of our study.

The 18 compounds in JEO were determined by GC-MS. Previous studies have found that limonene, β-myrcene, and linalool are present in the volatile oils of citrus fruit peels and inhibit Gram-negative bacteria (*Enterobacter aerogenes*, *Escherichia coli*), Gram-positive bacteria (*Bacillus cereus*), fungi (*Saccharomyces cerevisiae*, *Candida Albicans*) and viral (Gupta et al., 2021; Sevindik et al., 2021). The citrus essential oil contains γ-terpinene which inhibits the fungus *Alternaria*, *Hypocrea*, *Trichoderma*, and *Geosmithia* on pine wood, protecting it from discoloration (Martínez-Pacheco et al., 2022). Limonene, α-pinene, and β-pinene are the main components of the essential oil from *Campomanesia adamantium* (Cambess.) O. Berg leaves during flowering. It has high antibacterial activity against *Staphylococcus aureus*, *Pseudomonas aeruginosa*, and *Candida albicans* (Coutinho et al., 2009). *Foeniculum vulgare* Miller essential oil containing o-cymene, α-phellandrene, and α-pinene has anti-candida activity (Garzoli et al., 2018) α-terpineol present in Thymus vulgaris essential oil is a typical terpenoid that inhibits Penicillium fingertip resulting in damage to its cell membrane and cell wall (Jing et al., 2015). (-)-4-Terpineol is present in the essential oil of Melaleuca alternifolia and has inhibitory effects on *Aspergillus ochraceus* (Kong et al., 2019), yeast, and filamentous fungi (Pinto et al., 2014). These substances are also the main components of JEO, laying the foundation for its antimicrobial effect of JEO.

## Conclusion

In conclusion, this study revealed the *in vivo* and *in vitro* antifungal activity of JEO against gray mold on cherry tomatoes caused by *B. cinerea*. Moreover, JEO inhibits *B. cinerea* by disrupting the permeability of cell membranes, causing oxidative damage, altering mycelial morphology, and inhibiting pathogenesis-related enzyme activity. Therefore, JEO has the potential to prevent and control gray mold in cherry tomatoes and even other plant diseases.

## Data availability statement

The original contributions presented in this study are included in the article/supplementary material, further inquiries can be directed to the corresponding authors.

## Author contributions

Y-XW: methodology, investigation, and writing—original draft. Y-DZ: investigation, data curation, and writing—review and editing. NL: validation. D-DW: methodology. Q-ML: validation. Y-ZC: data curation. G-CZ and JY: resources, conceptualization, methodology, and supervision. All authors contributed to the article and approved the submitted version.
